# ‘I Condemn!’: A Discursive Analysis of Moral Condemnations in the Political Realm

**DOI:** 10.1007/s12124-025-09905-8

**Published:** 2025-03-31

**Authors:** Jonas Tellefsen Hejlesen

**Affiliations:** Department of Communication and Psychology, Aalborg University, Aalborg, Denmark

**Keywords:** Moral condemnation, Discursive psychology, Regulation of action, Moral responsibility, Political psychology

## Abstract

In this paper, I present a crude, provisional theory of moral condemnation based on a discursive analysis of an interaction between two prominent political figures – on the social media platform X (formerly known as Twitter) – in the aftermath of the Iranian missile strike on Israel on 1 October 2024. Based on the analysis, I argue that moral condemnations may serve as a tool for regulating action, and I provide a game-based analogy which may help encapsule two central aspects of moral condemnation: the construction of moral responsibility and a relationship of guilt *(setting the board)*; and the (attempt to) regulate action *(playing the game)*. Finally, I propose that we may also use moral condemnations as a substitute for action – especially in instances where the actor is either unable or unwilling to intervene. By morally condemning we may create a socially and/or personally legitimate excuse for inaction through a displacement of the responsibility to act – thus, ultimately allowing oneself to not do anything by not doing nothing.

*“You have stolen my dreams and my childhood with your empty words. And yet I’m one of the lucky ones. People are suffering. People are dying. Entire ecosystems are collapsing. We are in the beginning of a mass extinction*,* and all you can talk about is money and fairy tales of eternal economic growth. How dare you!”* (Greta Thunberg in NPR, 2019).

The topic of moral condemnation has gotten remarkably little attention from psychologists. Few studies have dealt explicitly with the phenomenon, and those that do focus primarily on the influence of specific factors on moral condemnation (e.g. Bourrat, Boumard, McKay & 2011; Cheng et al., 2013; Miller, Hannikainen & Kushman, 2014; Lucas, Galinksy & Murningham, 2016; Martin & Heiphetz, 2021). In those studies, the term is seldom defined and almost always used interchangeably with moral judgement (see also Mooijman & Van Dijk, 2015; Gamez-Djokic & Molden, 2016; Jarmakowski-Kostrzanowski & Radkiewicz, 2021; Jylkkä et al., 2021; Henderson & Schnall, 2021).^1^

Moral judgement and moral condemnation are however related but distinct concepts and treating the two as synonymous both reveals and leads to further conceptual confusion. The consequence of hereof is the neglect of a noteworthy and pervasive phenomenon that could otherwise be an important subject of study. To enable the possibility of such a study, the first step to be taken is to redress the conceptual confusion by clearly differentiating moral condemnation from moral judgement.

## A Definition of Moral Condemnation

An extensive definition is beyond the scope of this paper, so I will make it brief. Moral judgements are “(…) evaluations (good vs. bad) of the actions or character of a person that are made with respect to a set of virtues held to be obligatory by a culture or subculture” (Haidt, 2001 p. 817). ‘It is wrong to steal’, ‘Helping those in need is good’, and ‘We should take care of the planet’ are all moral judgements. They convey the message that a certain action is morally right or wrong, desirable or undesirable, acceptable or unacceptable.

In contrast, clear examples of moral condemnation regarding those same actions could be: ‘Thieves should be ashamed of themselves’, ‘Only a monster would refuse to help those in need’, and ‘No good person would ever show such little regard for the planet.’ These statements contain not only an assessment of the moral rightness or wrongness of the action but also express clear disapproval of the object of judgement. A working definition of moral condemnation might therefore be that it is *the act of expressing disapproval of something (an action*,* person*,* belief*,* organisation*,* practice*,* and so on) based on a negative moral judgement.*^2^  

## Political Condemnations

I stumbled into this topic during the ongoing 2023 Israel-Hamas war. At the time, the war had already spilled over into neighbouring Lebanon. Israel had just assassinated the secretary-general of Hezbollah, Hasan Nasrallah, and on 1 October 2024, just as I had begun writing, Iran launched around 200 ballistic missiles into Israel. In the days after the attack news outlets and social media sites filled with expressions of moral condemnation from world leaders, politicians, and political commentators. Such scenes of widespread moral condemnation are no rarity in the world of politics. When events occur – whether minor or major – that may be perceived as morally problematic, condemnations and/or calls for condemnations often follow. Anything from offensive statements and derogatory comments to terrorist attacks and military acts of aggression may give cause for condemnation.

From a social-psychological standpoint, we may raise a question concerning moral condemnations that was originally raised by Norman Whitfield and Barrett Strong regarding war: What is it good for? In other words, what is the function of such public expressions of moral condemnation? This is the central question that this paper aims to address – before attempting to sketch a crude, provisional theory of moral condemnation.

## Theoretical Framework

In this study, I draw extensively upon discursive psychology (see e.g. Edwards & Potter, 1992). From the perspective of discursive psychologists, the psychological domain is not internal structures or hidden inner processes; instead, we must look for psychological activities in discourse. Consequently, ordinary talk becomes a starting point for psychological investigation – as it is here that psychological phenomena are acted out (Potter, 2012, p. 104f). This points to two central ideas in discursive psychology that ground this study: (1) an insistence on “naturally occurring talk” as an apt object of psychological analysis (Potter, 1997); and (2) a focus on the actual use of language (i.e. function) rather than its referential meaning.

The discursive psychological framework shaped my engagement with the data in obvious ways. Most evident is the focus on language and how psychological activities are being done out in the open as people talk – rather than going beyond discourse and proposing explanations in the form of internal processes. Relatedly, it resulted in an engagement with what discursive psychologists call the ‘action orientation’ of speech - namely, what people do through their discursive acts rather than what they say.

## Method

As already mentioned, my investigation into this topic started when I stumbled upon an occurrence which provoked a sense of wonder. This is part and parcel of the abductive scheme of reasoning where one moves from unexpected observation to plausible explanation: (a) I observe something unexpected; (b) I question what might make this observation self-evident; (c) I suspect this explanation to be true (see e.g. Salvatore & Valsiner, 2010, p. 826; Brinkmann, 2014). In reasoning abductively, one does not aim to arrive at any set-in-stone truths – and the initial inference may very well show itself to be faulty. The explanation is only suspected to be true, and the inquiry continues in a never-ending process as proposed explanations are revised in light of new observations, which allows for the correction and/or expansion of flawed explanations.^3^ What it does aim for is a simple and plausible explanation for what was witnessed.

This provides the foundation for the single-case analysis carried out in this study. Through abductive inference, one moves from the particular to the general: from a single occurrence toward a possible generally applicable underlying set of rules which may explain that occurrence and other instances of it. This means that single cases are considered indispensable for knowledge generation as it is exactly in the particulars that general principles may be observed (see e.g. Valsiner, 2015).

### Data Collection

The study was based on 3 statements from prominent politicians posted on X (formerly known as Twitter) in the aftermath of the Iranian attack on Israel on 1 October 2024. All statements were posted from official X accounts and initially chosen based on the speaker’s role in the political world – as heads of state, leaders of world organisations, senior government officials, prominent figures in political parties, elected officials, and so on. An advanced search, which allows for further definition of search parameters (e.g., specific words or phrases, timeframe, engagements, etc.), was conducted.

The timeframe for the search was between 1 and 3 October 2024, as the attack was launched on 1 October 2024 and reactions usually occur within a day. The advanced search for posts contained the terms: ‘condemn’, ‘condemns’, or ‘condemned’ and filtered for replies and engagements. Replies were filtered so only posts, not replies, appeared in the search. Engagements were filtered to remove all posts with less than a hundred replies to remove noise (e.g. low-engagement posts and bot posts). This likely filtered out some relevant, but minor, posts. However, as the aim is not to conduct a qualitative analysis of all posts, but a quantitative study of a few, this is not a major issue.

The search generated 88 results of which 15 were unrelated and 32 were news or commentary. 41 posts from official X accounts of politicians, governments, and leaders of international organisations were obtained. All 88 posts were downloaded through exportcomments.com. From the 41 relevant posts, 2 were selected for in-depth analysis, along with a third post that fit the criteria, but did not show up due to a limitation in the advanced search function.^4^ The 3 posts were selected primarily because they comprise an interaction, thus giving a direct insight into the interpersonal and discursive nature of moral condemnations.

### Analytical Procedure

The analysis was done from a discursive psychological standpoint. A discursive psychological analysis cannot be executed by following a set of rigidly fixed steps. It is more like a craft skill (hence, the process eludes simple description). The analysis was, however, guided by Potter (2003) and Wiggins (2017). In analysing the posts, I paid careful attention to emotionally charged language (e.g. use of adjectives or morally loaded descriptors); inclusion or omission of identifiers; direct or indirect reference to someone/something; pronoun use; modal verbs; and sentences containing the words condemn, condemns or condemned and the surrounding context. I intentionally put very little focus on the word ‘condemn’ itself to avoid the assumption that merely stating the words ‘I condemn’ corresponds to doing it. The questions that guided me throughout the analysis were: Which actors appear and how are they constructed (in relation to each other)? What ‘account’ is being constructed?^5^ And what is being done with it – i.e. what function does it serve?

## Analysis

### Post 1: Antonio Guterres’ Condemnation


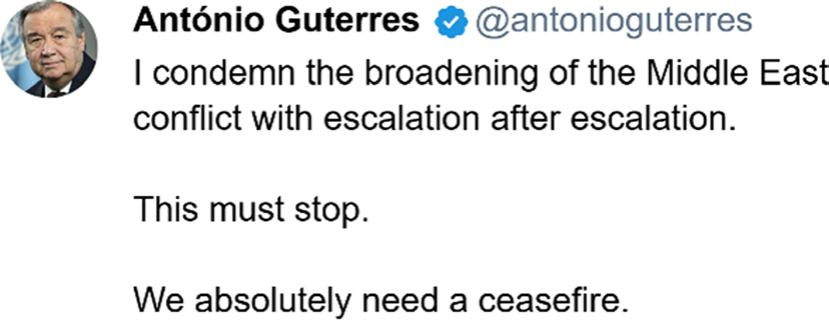


In this post, the Secretary-General of the United Nations, António Guterres, speaks not of the Iranian attack, but of “the broadening” of the conflict. In doing so, he describes the attack as an exacerbation of an already existing conflict by directing attention not just to this specific attack, but also to events leading up to it. While he calls the attack an “escalation”, it is an escalation in a “conflict with escalation after escalation.” In phrasing the situation such, he does several important things: Firstly, he explicitly situates the attack within a broader context, implying that the conflict is not one-sided and that the attack did not appear out of nowhere – it was not an initiation but a “broadening”, an “escalation”. Secondly, he (verbally) equates the attack with prior attacks in the conflict by classifying them under the same label of “escalations”, thus attributing responsibility to both sides.

It is worth noting, however, that the actors responsible for “escalation after escalation” are not explicitly mentioned. A possible explanation is that, by doing so, Guterres constructs a story of prospective responsibility rather than one of attribution of blame. It is not about who did what, but about what can be done to avoid further escalations. In that light, “This must stop” is not merely an expression of his disapproval, but this is also an appeal to the actors: it is a necessity that the conflict does not continue! This necessity is further stressed in the final utterance: “We absolutely need a ceasefire.” It is not a want, but a need. And in making a shift from the initial “I” to a “We”, Guterres further cements that this need is shared. While this “We” is ambiguous – and can refer to the United Nations, it is equally possible that it refers to humanity in general; in which case, the need for a ceasefire is presented as an absolute and universal *human* need.

To summarise: In refusing to explicitly focus on Iran as the perpetrator and Israel as the victim, and instead attributing responsibility to both parties, Guterres refrains from siding clearly with one or the other. Instead, he tries to position himself between the two, ‘preparing the ground’ for a dialogue between the two parties – with him as a possible intermediary. He appeals to a common denominator – their shared humanity – which can unite them inside a shared community within which both can choose to exist but currently do not. In condemning the escalations of both sides – acts which undermine the common moral order of the shared community–, he positions them both as transgressors, existing on the outskirts of this shared community, while simultaneously creating the possibility for a re-entrance into it.

### Post 2: Israel Katz’s Response To Guterres

Consequently, several responses were made from (people within) the Israeli government in reaction to Guterres’ statement. The most interesting (and most viewed) response came from the Israeli Minister of Foreign Affairs, Israel Katz.


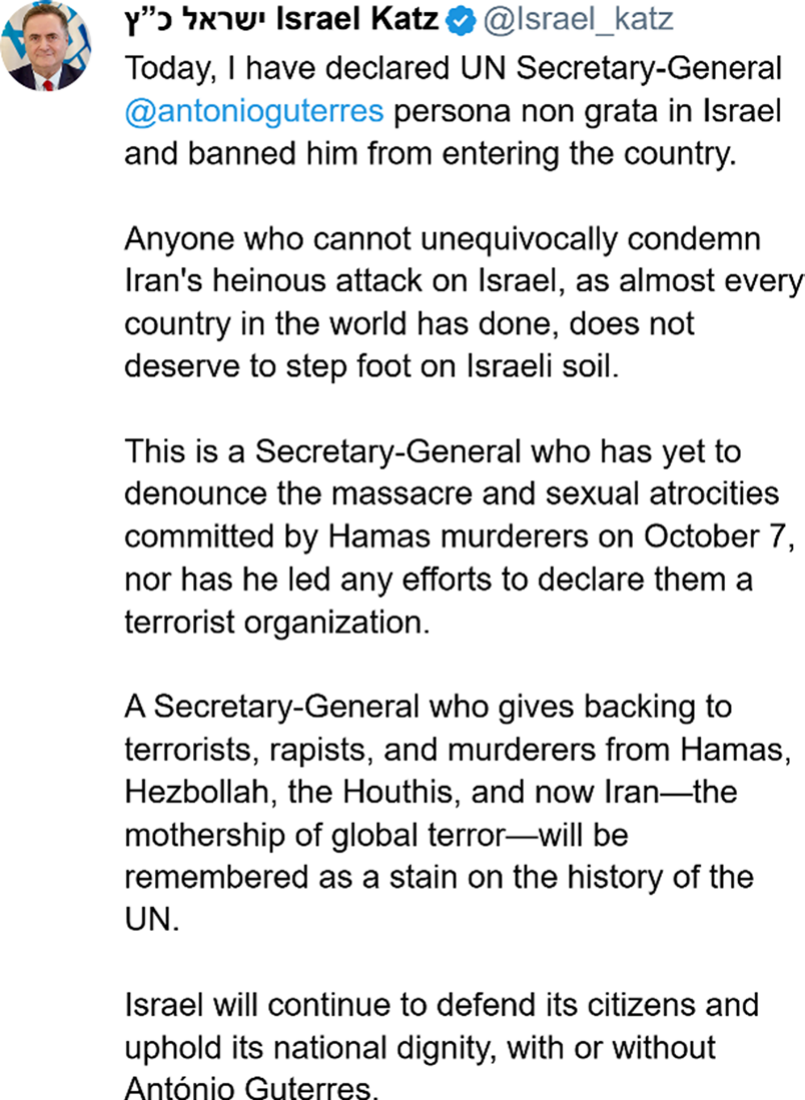


In his response, Katz discloses that he has declared Guterres *persona non grata*. A statement that is not just a simple conveyance of information, but which, as the following lines reveal, serves to make an example of Guterres. It is not just Guterres who – because he failed to “unequivocally condemn Iran’s heinous attack on Israel” – does not “deserve to step foot on Israeli soil.” It is “anyone” who fails to do the same. With this phrasing, Katz draws a sharp line: you are either with us or against us. And by asking for “unequivocal” condemnation and showing that those who fail are punished, he displays that there is no room for ‘bothsidesism.’

Furthermore, Katz paints Guterres’ character in a particular light: He is not just a person who failed to unequivocally condemn this attack – a moral failing made even more significant considering “almost every country in the world” managed to do so. He is also someone who has failed to “denounce the massacre and sexual atrocities committed by Hamas murderers on October 7” and to lead “any efforts to declare them a terrorist organization.” And so, he is presented as a supporter of “terrorists, rapists, and murderers” and “the mothership of global terror” – and thus, a man who “will be remembered as a stain on the history of the UN.” Interestingly, a shift from a definite article, “the Secretary-General”, to an indefinite article, “a Secretary-General” occurs. In doing this, Katz’s attack is less direct and personal. It is not an attack on Antonio Guterres per se; any secretary-general who acted so would be judged the same.

Lastly, Katz constructs Israel as a country that – in stark contrast to Iran and their allies – merely “defend[s] its citizens and uphold[s] its national dignity” – and in doing so, he does not simply assert their right to defend themselves, but he also rejects Guterres’ equalisation of the actions of Israel with the actions of their enemies.

To summarise: in this post, we again see how certain actors are positioned in relation to a moral order: In describing Hamas, Hezbollah, the Houthis, and Iran as “terrorists, rapists, and murderers”, Katz firmly characterises them as actors who exist outside the moral community. In contrast, he rejects Guterres’ portrayal of the events and categorises Israel as morally righteous in simply defending its citizens against the monsters who attack them. And due to his failure to properly condemn these monsters, Guterres is placed on the side of the former rather than the latter – on the side of evil rather than good; outside the moral order rather than within it.

### Post 3: António Guterres Response To Katz

Before continuing with the final response, a further remark should be made on the analysis above. Some of the passages can be interpreted as leaving the door open for Guterres to re-enter the moral order – thus placing him on the border, rather than firmly outside it. Firstly, the shift from the definite, “the Secretary-General” to the indefinite, “a Secretary-General”, can also be interpreted as a subtle hint that Guterres does not need to become a “stain on the history of the UN” – that, while he is about to become that, he can avoid it if he sides with Israel and unequivocally condemns Iran. This interpretation can be backed further by the final line, “*with* or without Guterres” which might also be seen as an appeal to Guterres to come to Israel’s side.^6^ And seemingly, the response did not fall on stony ground:


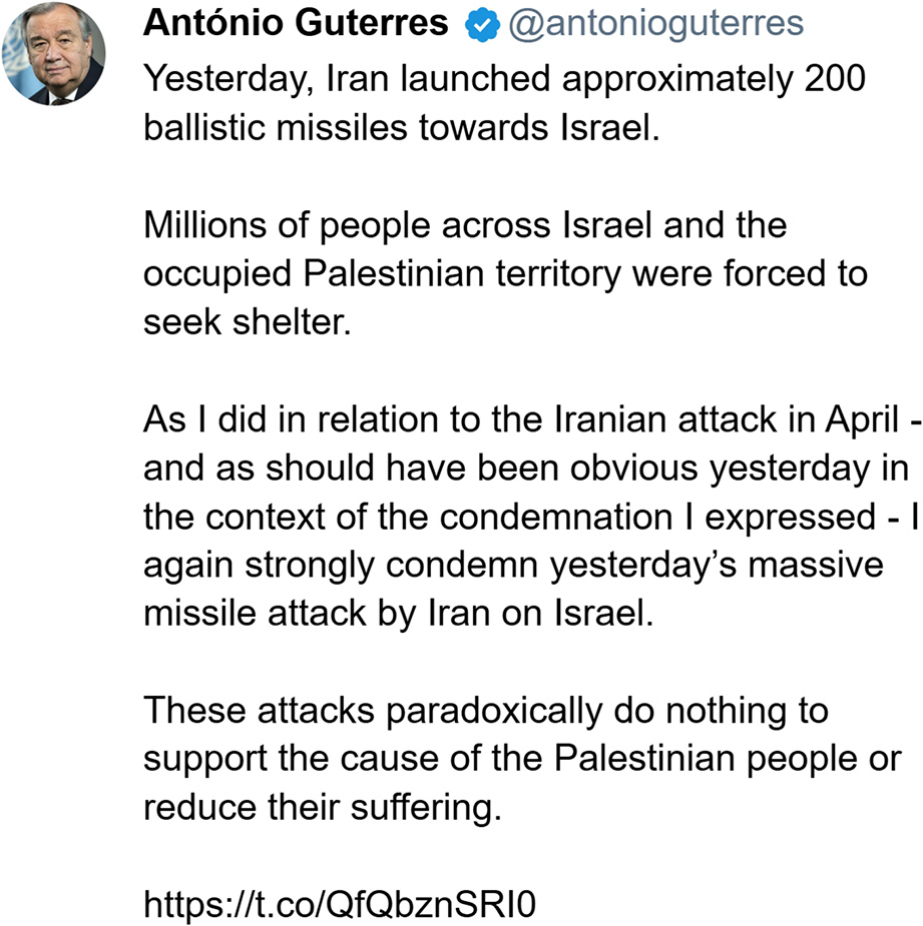


While much can be said about this post, I will keep it brief by focusing on the following lines: “As I did in relation to the Iranian attack in April – and as should have been obvious yesterday in the context of the condemnation I expressed – I again strongly condemn yesterday’s massive missile attack by Iran on Israel.” In this sentence, he constructs his prior record in quite a different way than Katz did and thus repudiates the characterisation of him as morally corrupt. Rather than being a Secretary-General who “gives backing to terrorists, rapists, and murderers”, he is a Secretary-General who condemned the Iranian attack in April and has no issue in strongly condemning this attack (again) either. In further refutation of Katz’s unfavourable characterisation, he also refuses to accept the Israeli interpretation of his previous post: He does not apologise for failing to properly condemn the attack but reiterates that he “again strongly condemn(s)” the attack. Furthermore, he constructs the whole thing as a misunderstanding brought about not because he failed to convey his intention properly, but because of a failure of others to understand what “should have been obvious in the context.”

Following up on the ambiguity of Katz’s post, Guterres’ post can be seen as both a (partial) rejection of the condemnation and a (reluctant) acceptance of it. In refusing to accept Katz’s version of events and portrayal of him, he rejects the condemnation (treating it as unfounded, based on a false conception of reality). On the other hand, in changing his conduct, he seemingly accepts the condemnation. And so, it seems that, perhaps, the two are not entirely mutually exclusive: Guterres (explicitly) rejects Katz’s condemnation, but at the same time, he changes his conduct (thus implicitly accepting it) – all the while insisting that nothing has changed.^7^  

## Discussion

In light of the analysis above, we can see two central aspects of moral condemnations: First is a sort of ‘moral mapping’ in which we (often implicitly) construe others in a certain way in relation to a moral community (within it, on the border, outside of it). A central part of this is the construction of a ‘*Schuldverhältnis’*^8^ (based on actions or association) which serves to both construct and justify the map. Second is the (attempt) to regulate action (based on the moral map) – i.e. inciting someone to act in a certain way. These two aspects can be captured with the game analogy of *setting the board* and *playing the game*. In the same way, you need to set the board before you play a board game, ‘moral mapping’ sets the board. And once the board has been set, it becomes possible to ‘play the game’ – that is, to attempt to regulate action (which is seemingly a primary function of moral condemnation).

However, if anything is clear from the analysis above, it is that there may very well be disagreement regarding the ‘positions of the pieces on the board.’ This can lead us to think that, in the above, ‘the game’ is not even being played yet: We are not watching people play chess. We are watching them dispute over where the different pieces are supposed to be! We should, however, not let this fool us. To save the game analogy, it is necessary to stress that the game in question is more like battleship than chess in the sense that (1) there need not be agreement on the position of the pieces and (2) the setting of the board has a bearing on the game; it is, in other words, a part of the game.

This is an important observation which may give us some insight into the success or failure of a condemnation. It seems that, for regulation, there must be some degree of agreement on the pieces’ position on the board – to stick with the game analogy. If there is too large a discordance, the dispute remains on the setting of the board – and so, the disagreement is on the placement of the moral responsibility (as in the case of Katz’s response to Guterres). If, however, there is considerable agreement, and the ‘moral map’ is accepted to some degree the focus becomes on how the parties may live up to their respective moral responsibilities.

To elaborate further here, we see also how, while the object/target of condemnation can be the intended audience of the condemnation, it is not always so – in the same way that in a debate, it is not always the mind of the opponent, but sometimes the mind of the audience that one is trying to change. In those instances, the condemner addresses others (whose moral map coincides somewhat with themselves) to mobilise them to action against the object of condemnation.

Relatedly, there seems to be some consistency concerning who is ‘addressable’ and who is beyond reach. As mentioned in passing, it seems that (in condemning) we place actors within, on the border of, or outside the moral community. The condemner is always within the moral community, while the condemned can both be outside it or on the border. Actors within the moral community are beyond reproach. They are the regulators, not the regulated. Actors outside the moral community are beyond reach. They are the object of condemnation, but not the audience; they are so far outside the moral community that the condemner sees no possibility of return/redemption. Actors on the border of the moral community are within reach. They are both the object and the audience of the condemnation; though they have transgressed the moral order, they are still redeemable – if they accept their moral responsibility and change their behaviour.

### Condemnation as a Substitute for Action

So far, it has been argued that moral condemnation can be a tool for regulating action. It is also possible, however, to view it as a substitute for action; a tool which enables inaction by simulating action in inciting others to act (in one’s place) – such that while I am not doing something, I am also not doing nothing.

Israel, for example, did not express any condemnations of Iran; they simply retaliated. Where one punishes, one need not condemn. On the other hand, however, the (leaders of) countries who condemned Iran, did not retaliate. And so, it seems, the reverse applies as well: where one condemns, one need not punish. In this way, condemnation can serve as a substitute for action. Where a moral transgression occurs, an urge to punish (or at least an expectation of punishment being meted out *by someone*) often emerges. By condemning, we may relieve the pressure to act (whether it is felt internally, due to external expectations, due to an anticipation of expectations, and so forth) and allow inaction – without the sense (or charge) that one is shunning one’s moral responsibility.

Condemning, then, signals a recognition of the transgression and a belief in a need for redress while (dis)placing the responsibility to act elsewhere – thus, creating a socially and/or personally legitimate excuse for inaction.

A final point should be added here, regarding the significance of power. I began this paper with an epigraph quoting Greta Thunberg’s (in)famous address at the 2019 UN Climate Action Summit. Thunberg is disturbed by a world at “(…) the beginning of a mass extinction” where those in power continue to talk only about ”(…) money and fairytales of eternal economic growth” (NPR, 2019). And while Thunberg may have the social power to bring attention to the issues, she is politically powerless – she has no means of directly enforcing change. Thus, standing powerless before the embodiment of global power, her last resort is to condemn – and so, appeal to those who do have the power (and encourage others to follow in her footsteps). Condemnation, in this instance, may be seen as a ‘cry of powerlessness.’^9^ It is paradoxically an exertion of power through the admittance of powerlessness: Here I condemn; I can do no other.^10^

## Data Availability

Full transcripts of analysed data included in the article.

## Footnotes

1 Instances of this can also be found in studies on moral judgement (e.g. Forsyth, 1985; Haidt, 2001; Schnall et al., 2008; Schein & Gray, 2018). However, there are as many, if not more, examples of studies on moral judgements where the word ‘condemnation’ does not occur at all (e.g. Rest, 1973; Miller & Bersoff, 1992; Nichols, 2002; Cushman et al., 2006; Amit & Greene, 2012).

2 ‘Pure’ moral judgements are however rare in our everyday lives. People seldom express that something is wrong without also (overtly or covertly) morally condemning that which they judge as wrong. Saying ‘lying is wrong’, for example, is often more than merely expressing a moral evaluation of the action of lying. It is like saying ‘I disapprove of lying – not because this is my subjective moral judgement but because lying is objectively morally wrong. Stop lying!’ From this, it follows that studies on moral judgement often provoke moral condemnations along with moral judgements. But while these studies focus on our conceptions of moral rightness or wrongness of certain things and in what it is grounded, studies on moral condemnation would focus specifically on the expression of disapproval – its function, effects, mechanisms, and so on.

3 Here it is also worthwhile to mention that in reasoning abductively, we bring certain plausible interpretations among many - which may allow us to make sense of the phenomenon - to the forefront. This provides a lens through which it becomes possible to make sense of the phenomenon under investigation - but a lens to be discarded or refitted when it no longer sharpens our sight.

4 The post fits the search criteria as outlined above wherefore it should have appeared. However, if a post exceeds 280 characters and the search terms do not appear within the first 280 characters, it does not appear in the advanced search. I found the post (which is a response to the second post) as I suspected a response would have been made, and so, I went looking for it.

5 To give a very basic example: saying that someone was ‘randomly attacked’ brings forth a very different account than saying that they ‘got what they deserved’. Each account smuggles a certain portrayal of events with them.

6 Although, an equally – or maybe more - plausible interpretation is that this is less an invitation to cooperate than a threat (that they will continue independently of his cooperation).

7 This ambiguous reaction may be interpreted as a sign of shame. In denying the wrongdoing, getting defensive, and attempting to redress the wrong all at once, there is an ongoing negotiation of shame (and moral responsibility). There are limits to the wrongdoings which we can admit to (without shattering our I-ideal; or, more cynically, our public image); thus, it is necessary for Guterres to diminish (and even deny) his wrongdoing before it becomes possible for him to then accept it.

8 Here, I use a German phrase (equivalent to the Danish word *skyldsforhold*). *Verhältnis *translates as *relationship* and *Schuld *may mean *fault*,* guilt*, or *debt* depending on the context. I use the German word as I cannot conceive of an English term which retains those slight nuances; the closest is perhaps ‘relationship of guilt’ or ‘relationship of (moral) indebtedness’, but neither really hit the mark.

9 Note that this also means that (otherwise) powerful political figures may declare their lack of power by expressing moral condemnations.

10 Naturally, power is not the only factor – as already established. One needs both the means, the will, and (in some instances) the popular support. Actors who have both the means and the will (and the popular support) to punish need not condemn. Actors who do not have either the means or the will (or the popular support) may find themselves restricted to condemnation – and hoping for intervention from elsewhere. In not having the means, they may, by condemning, attempt to gain the attention (and sympathy) of those who do. In not having the will, they may, by condemning, resolve themselves of responsibility (and any charges of moral shortcomings and/or feelings of guilt, shame and the like). And in not having the popular support, they may, by condemning, whip up support for the cause.
